# An outbreak after all: *Cutibacterium acnes* among pediatric patients with cerebrospinal fluid diversion device infections highlights gaps in guidelines

**DOI:** 10.1017/ash.2024.359

**Published:** 2024-09-12

**Authors:** Felicia Scaggs Huang, Cameron Griffin, Matthew Fenchel, Melanie DuBose, Andrea Ankrum, Joshua K. Schaffzin

**Affiliations:** 1 Department of Pediatrics, University of Cincinnati College of Medicine, Cincinnati, OH, USA; 2 Division of Infectious Diseases, Cincinnati Children’s Hospital Medical Center, Cincinnati, OH, USA; 3 Deloitte, Cincinnati, OH, USA; 4 Division of Biostatistics and Epidemiology, Cincinnati Children’s Hospital Medical Center, Cincinnati, OH, USA; 5 James M Anderson Center for Health Systems Excellence, Cincinnati Children’s Hospital Medical Center, Cincinnati, OH, USA; 6 Infection Prevention & Control Program, Cincinnati Children’s Hospital Medical Center, Cincinnati, OH, USA; 7 Division of Infectious Diseases, Immunology, and Allergy, Children’s Hospital of Eastern Ontario, Ottawa, ON, Canada; 8 Department of Pediatrics, University of Ottawa, Ottawa, ON, Canada

## Abstract

**Objective:**

*Cutibacterium acnes* is normal skin flora but can cause sterile implant infections. We investigated a cluster of seven patients with *C. acnes* in anaerobic cerebrospinal fluid (CSF) cultures in November 2020. Further analysis identified a missed outbreak, highlighting ambiguity in diagnosis of indolent organisms in the 2017 IDSA meningitis guidelines.

**Design:**

Outbreak investigation.

**Setting:**

Quaternary pediatric facility.

**Patients:**

A case was defined as a hospitalized patient with *C. acnes* isolated from CSF culture from January 1, 2016 to December 31, 2022.

**Methods:**

We defined comparison periods based on timing of *C. acnes* culture positivity as 1) pre-outbreak (2016–2020), 2) outbreak (2020–2021), and 3) post-outbreak (2022). Rates of *C. acnes* positive cultures per 1000 CSF cultures and rate ratios were calculated by comparison periods.

**Results:**

We identified 9 positive *C. acnes* CSF cultures among 7 cases November 10–27, 2020, all with at least 1 CSF diversion device. The anaerobic culture media was substituted at the time of case cluster. In 2021, the culture media was implemented permanently with no increase in *C. acnes* culture positivity. The rate of *C. acnes* positive CSF cultures and rate ratio increased in the outbreak period (p=0.01) compared to pre-outbreak and post-outbreak periods. There was no difference between the pre- and post-outbreak periods.

**Conclusions:**

Retrospective analysis of CSF culture data led to reclassifying a *C. acnes* pseudo-outbreak as a true outbreak in CSF diversion devices at our institution. Clearer guidance is needed to delineate the role of *C. acnes* in CSF diversion device infections.

## Introduction

*Cutibacterium acnes* is an anaerobic, Gram-positive bacillus and normal component of the skin microbiome. *C. acnes* is implicated in infections of sterile implants such as cerebrospinal fluid (CSF) diversion devices.^1^ Presentations can be subtle, with minimal CSF abnormalities or clinical symptoms, and differentiating true infection from sample contamination is challenging.^2^ As a low-pathogenicity skin-colonizing organism, *C. acnes* may be classified clinically as a contaminant not requiring treatment, particularly if it grows in broth culture only or from cultures whose sterility is unclear which could result in a delay in clinical care or deferred initiation of an outbreak investigation.^3,4^ Infectious Diseases Society of America (IDSA) guidelines recommend device removal and antimicrobial therapy when multiple consecutive positive cultures or a clinical picture consistent with infection are identified.^2^ However, there is also limited low-quality evidence for outcomes of *C. acnes* shunt infection.^5,6^ While *C. acnes* can cause CSF diversion device infection, the optimal approach to differentiate infection from contamination is not straightforward.

We initially investigated a cluster of seven hospitalized pediatric patients with *C. acnes* isolated from anaerobic cultures of CSF at a quaternary children’s hospital in November 2020. We declared a pseudo-outbreak due to use of a potentially more sensitive anaerobic culture media during October and November 2020. However, we later hypothesized that we failed to detect a true *C. acnes* outbreak and missed the opportunity to identify the etiology. We conducted a review of our experience to assess the impact of the IDSA guidelines on the diagnosis of indolent pathogens like *C. acnes* in the CSF of all patients to inform our response to similar future situations.

## Methods

### Case definition

A case was defined as a hospitalized patient with *C. acnes* isolated from a CSF culture. During the investigation, we collected case demographic data from the electronic medical record (EMR) including age, sex, admission date, hospital unit at time of culture, CSF diversion device type, surgeon performing device placement and date, positive culture date, individual who collected positive sample and collection method, response to positive culture (such as antibiotic therapy and surgical intervention), and discharge date. For our expanded investigation of pre- and post-outbreak periods, we collected the same fields as the outbreak period and additionally assessed antimicrobial therapy, surgical intervention, and if an infectious diseases consultation was obtained. We used an enterprise intelligence resource (Vigilanz Corp, Minneapolis, MN) to obtain CSF culture numerator and denominator data.

### Outbreak investigation

When Infection Prevention & Control (IP&C) determined an increase over baseline of *C. acnes* culture positivity in CSF, a multidisciplinary investigation team was formed that included IP&C, Microbiology, and Neurosurgery. Assessment of all relevant processes such as CSF collection, CSF culture incubation and analysis, and routine care of CSF diversion device was conducted via stakeholder interviews. No company or Food and Drug Administration statement reported an increase in *C. acnes* isolation from the media. No *C. acnes* clusters or outbreaks were reported from other institutions

### Statistical analysis

We defined comparison periods based on the timing of our *C. acnes* culture positivity increase as: 1) pre-outbreak (January 1, 2016 to October 31, 2020, 2) outbreak (November 1, 2020 to December 31, 2021), and 3) post-outbreak (January 1, 2022 to December 31, 2022).

Rates of *C. acnes* positive cultures per 1000 CSF cultures were calculated by 1) all anaerobic and aerobic cultures combined, 2) all anaerobic cultures, and 3) all aerobic cultures during each of the 3 comparison periods. For patients with multiple positive cultures, we defined a new event as a positive culture at least 14 consecutive days after the previous positive regardless of interim positive cultures.

For patient characteristics, means and standard deviations (with range) were calculated for continuous variables; counts and percentages for categorical variables. Using log-offset, zero-inflated Poisson models for rates, we tested the effect of time in months on unique patient count and incident events. Rate ratios were calculated for comparison periods.

## Results

### Outbreak investigation

**Figure 1.**
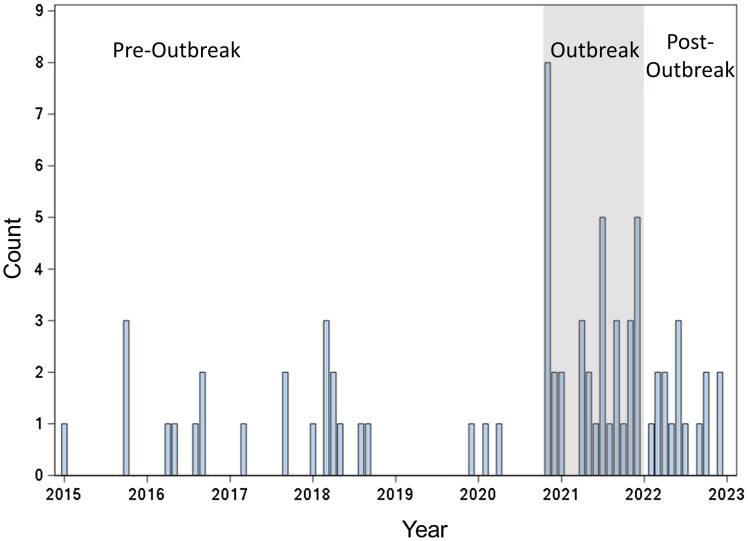
Epidemic curve of monthly *C acnes* positive CSF cultures 2015–2023. The outbreak period is denoted by the shaded box.

We identified nine CSF cultures positive for *C. acnes* among seven cases during November 10-27, 2020 (Figure 1). All had at least one CSF diversion device placed prior to sample collection, and all had a diversion device in place at the time of collection. Clinical protocols for CSF collection and microbiologic analysis are standardized and have not changed. All staff permitted to collect samples were required to demonstrate competency during their onboarding process. Routine anaerobic cultures were added to all neurosurgical device samples in 2018 and all CSF samples in fall 2020 per updated IDSA and Clinical & Laboratory Standards Institute (CLSI) guidelines,^2,7^ respectively. Neurosurgical providers and laboratorians were notified of the cluster and protocol adherence was reinforced.

The anaerobic fluid thioglycollate culture media that had been in use in the pre-outbreak period (Becton, Dickinson and Company, Sparks, MD) was substituted at the same time as our case cluster (Anaerobe Systems Inc., Morgan Hills, CA, late October/early November 2020). There were no significant substitutions for anaerobic solid media products. No further cases above baseline rates were identified after November 2020.

### Patient demographics

During the outbreak period, the seven affected patients had a median age of two months, 71.4% of patients had a single culture positive for *C. acnes,* and 42.8% required no medical or surgical interventions (data not shown). On our review of patients with *C. acnes* in CSF cultures at our institution across all periods (January 1, 2016 to December 31, 2022), we noted that the mean patient age was 8.9 years, 32.4% of patients received antimicrobial therapy, 22% underwent surgical intervention, and 57% received an infectious diseases consult (Table 1). Ceftriaxone was the most commonly administered antibiotic (29.7%, 11/37) and 48.7% were treated for >10 days (Table 1).

**Table 1.** Characteristics of patients and cerebrospinal fluid (CSF) cultures with isolated *Cutibacterium acnes* and therapies during study period

Characteristic	Frequency (n)
Unique patients with identified *C. acnes*	65
Age (mean)^a^	8.9 years (range 2 days to 25.0 years)
Male sex^a^	35 (54%)
Days to culture positivity (mean)	8.2 (range 3 to 15 days)
Number of positive cultures	
1	55 (84.6%)
2	7 (10.8%)
≥3	3 (4.6%)
Surgical intervention^b^	16 (22%)
Infectious diseases consult^b^	42 (57%)
**Antibiotics** ^b^	
Any	24 (32.4%)
1	22 (29.7%)
≥2	2 (2.7%)
Antibiotic agent^c^	
Amoxicillin	1 (2.7%)
Ampicillin	1 (2.7%)
Cefepime	3 (8.1%)
Ceftazidime	1 (2.7%)
Ceftriaxone	11 (29.7%)
Clindamycin	1 (2.7%)
Doxycycline	5 (13.6%)
Penicillin G	7 (18.9%)
Vancomycin	7 (18.9%)
Antibiotic route^c^	
Enteral	7 (18.9%)
Intravenous	29 (78.4%)
Unknown	1 (2.7%)
Antibiotic treatment duration^c^	
≤3 days	9 (24.3%)
4–6 days	0 (0%)
7–10 days	10 (27.0%)
11–14 days	7 (18.9%)
>14 days	11 (29.8%)

a Statistics for age and sex based on unique patients, first encounter (n = 65).

b Percentages for surgical intervention, infectious diseases consult, and antibiotics based on positive encounters (n = 74).

c Percentages for antibiotic agent, route, and duration based on total encounters receiving antibiotics (n = 37).

### C. acnes culture events and rates

For the study period, there were 69 CSF cultures positive for *C. acnes* in 65 patients among 24,420 cultures obtained (Figure 1). The mean number of days from CSF sample collection to culture positivity was 8.2 days with the vast majority of patients having a single positive culture (84.6%, Table 1).

The seven events noted in November 2020 were an increase over the prior and following six months. The rate of positive anaerobic CSF culture for *C. acnes* was higher during the outbreak period (November 2020–December 2021, 33 per 1000 cultures) compared to the pre-outbreak (January 2016–October 2020, 13 per 1000 cultures) and post-outbreak periods (January-December 2022, 14 per 1000 cultures) (Table 2). The rate ratio during the outbreak period was also increased relative to both the pre-outbreak (RR 2.7, p = 0.01) and post-outbreak (RR 2.3, p = 0.01) periods (Table 3). The *C.* acnes rate of aerobic and anaerobic culture positivity increased from 5.7 to 14.2 per 1000 CSF cultures from the pre-outbreak and post-outbreak periods, though this did not achieve statistical significance (RR 1.1, p = 0.7) (Tables 2–3).

**Table 2.** Rate^a^ of positive *Cutibacterium acnes* cultures in the cerebrospinal fluid (CSF) per 1000 CSF cultures from January 1, 2016 to December 31, 2022

Time period^b^	*C. acnes* rate of anaerobic and aerobic cultures* (95% CI)	*C. acnes* rate of anaerobic cultures* (95% CI)	*C. acnes* rate of aerobic cultures* (95% CI)
Pre-outbreak period	5.7 (3.3–9.9)	12.6 (6.3–25.2)	2.5 (1.4–4.6)
Outbreak period	27.0 (18.4–39.6)	33.4 (22.9–48.8)	2.9 (0.9–9.1)
Post-outbreak period	14.2 (7.9–25.5)	14.5 (8.3–25.1)	Not applicable^c^

CI, confidence interval.

a Rate calculation based on number of positive *C. acnes* cultures of 1000 CSF cultures.

b Comparison periods are defined as pre-outbreak (January 1, 2016–October 31, 2020), outbreak (November 1, 2020–December 31, 2021), and post-outbreak (January 1, 2022–December 31, 2022).

c Estimates for aerobic cultures for the post-outbreak period are not included as there were no positive aerobic cultures.

**Table 3.** Rate ratio of positive *Cutibacterium acnes* anaerobic cultures in the cerebrospinal fluid (CSF) by time period^a^ from January 1, 2016–December 31, 2022

Time period comparison^a^			RR	95% CI	*p-value*
Pre-outbreak period	vs	Outbreak period	2.7	1.2–5.7	0.01
Post-outbreak period	vs	Outbreak period	2.3	1.2–4.4	0.01
Pre-outbreak period	vs	Post-outbreak period	1.1	0.5–2.7	0.7

a Comparison periods are defined as pre-outbreak (January 1, 2016–October 31, 2020), outbreak (November 1, 2020–December 31, 2021), and post-outbreak (January 1, 2022–December 31, 2022).

## Discussion

We declared our 2020 case cluster a pseudo-outbreak based on a hypothesis that a culture media change improved detection of *C. acnes* in CSF at our institution and thus represented false-positive results. However, a stable *C. acnes* CSF culture rate after the media was changed permanently suggests the case cluster represented a true outbreak. Misclassification of the cluster led to a missed opportunity to identify the actual root cause of the outbreak and prevent future cases.

Pediatric CSF diversion device infections occur in up to 10% of patients depending on underlying risk factors and reinfection rates have been reported as high as 20%.^8,9^ The proportion of infections caused by *C. acnes* is poorly defined (1.4%–15% in some series).^10,11^ The variability in diagnosing a *C. acnes* infection may be due to absence of typical symptoms and relatively slow growth of the organism in culture.^12,13^ Although the 2017 IDSA guidelines recommend incubating CSF cultures for up to 10 days to promote growth opportunity of *C. acnes*, ambiguity remains whether this intervention would identify additional *C. acnes* infections or would increase the false-positivity rate of CSF cultures as most of our cultures resulted in <10 days.^2^

The clinical intervention for neurosurgical CSF diversion device infection involves extended hospitalization and immobilization for external CSF drainage, and prolonged antibiotic courses often requiring peripherally inserted central catheter (PICC) placement.^14,15^ Revision of a neurosurgical shunt for any indication increases the risk of subsequent infection that rises with each additional revision.^16^ Inaction may not be benign as delayed diagnosis of neurosurgical shunt infections can have significant impact on patient outcomes, resulting in seizures, cognitive impairment, and death.^17,18^ Thus, the decision to revise a neurosurgical CSF diversion device is far from trivial and introduces risks that are justifiable for high-pathogenicity organisms but may be less clear for low-pathogenicity organisms like *C. acnes*.

In this ambiguous context, we investigated the 2020 case cluster. The incidence of positive cultures was elevated, but the incidence of true infection was less clear. Clinical management of the seven patients was predominantly with observation. Only two of the seven cases were treated with antibiotics and one had surgical intervention although six had an infectious diseases consultation (data not shown). This suggested that clinical teams considered these positive cultures clinically insignificant and may have biased us to conclude we had a pseudo-outbreak.

Changes in anaerobic culture product use in the Microbiology Lab allowed us to reexamine our conclusion. We were surprised that *C. acnes* culture-positive rates returned to pre-outbreak levels and concluded that our 2020 cluster was indeed an outbreak. To understand impact on patients and utilization, as well as inform future responses to similar clusters, we reviewed charts of all patients with a *C. acnes*-positive CSF culture from 2016 to 2022. There was considerable variation in management, with the majority of cases receiving no interventions (Table 1).

Upon review, we concluded our surveillance system appropriately detected an increase in *C. acnes* cases, which is its goal. We faltered in our further analysis, however, when we considered the likelihood of case finding with a low-pathogenicity organism that is a ubiquitous colonizer. We did not pursue investigation tools such as molecular analysis of the isolates. However, should future clusters occur, we may include them earlier in the process in these high-risk populations even if the epidemiologic link is unclear to identify a source.^19^ Thankfully, the outbreak self-resolved, but we cannot be sure the issue causing it was remediated.

We also concluded that guideline ambiguity around diagnostic criteria for infection likely contributed to our failure to recognize the outbreak. Contrary to IDSA guidance, holding our cultures for 10 days had little impact on our investigation or clinical care. Similarly, multiple consecutive positive cultures helpful for clinical decision-making would not influence the threshold to initiate outbreak investigation. Future guidelines may consider more stringent criteria to define true infection for *C. acnes* or other low-pathogenicity organisms. Guidance for oral suppressive regimens in patients who may not be good candidates for surgical intervention could support clinician decision-making and avoid surgical risks. Ultimately, greater guidance clarity would enable institutions to better discern device-associated outbreaks of indolent organisms like *C. acnes* earlier and intervene more effectively.

Our study is subject to limitations. Our delineation of pre-outbreak, outbreak, and post-outbreak time periods is arbitrary and may not represent optimal categorization. We had 12 months of post-outbreak data and are likely insufficiently powered to determine a difference. Since we observed a difference with the time periods chosen, shortening the outbreak period would likely generate higher infection rates and lead to a similar result. Additionally, we could not account for all potential changes in sample collection personnel or protocols in the pre-outbreak period. Nonetheless, our protocols have been standard in our institution for years, and many team members have had long tenures.

## Conclusions

Retrospective analysis of CSF culture data led to reclassifying a *C. acnes* pseudo-outbreak as a true outbreak in CSF diversion device infections at our institution. Further analysis highlighted that the indolent nature of the organism and ambiguity of current guidelines can hinder effective determination of an outbreak. As the field evolves, hospital epidemiologists in resource-rich settings may utilize molecular analyses more routinely to evaluate for a point source, even if the epidemiologic link is unclear. Additionally, more studies and clearer guidance are needed to delineate the role of *C. acnes* in CSF diversion device infections and the optimal approach to diagnosis and treatment.
